# Genotypic and phenotypic drug resistance patterns of *Mycobacterium tuberculosis* isolated from presumptive pulmonary tuberculosis patients in Ethiopia: A multicenter study

**DOI:** 10.1371/journal.pone.0303460

**Published:** 2024-05-16

**Authors:** Bazezew Yenew, Abebaw Kebede, Ayinalem Alemu, Getu Diriba, Zemedu Mehammed, Misikir Amare, Biniyam Dagne, Waganeh Sinshaw, Ephrem Tesfaye, Dereje Beyene, Woldaregay Erku Abegaz

**Affiliations:** 1 Ethiopian Public Health Institute, Addis Ababa, Ethiopia; 2 Department of Microbiology, Immunology and Parasitology, College of Health Sciences, Addis Ababa University, Addis Ababa, Ethiopia; 3 Department of Microbial, Cellular and Molecular Biology, College of Natural and Computational Sciences, Addis Ababa University, Addis Ababa, Ethiopia; 4 Africa Centres for Disease Control and Prevention, Addis Ababa, Ethiopia; Yekatit 12 Hospital Medical College, ETHIOPIA

## Abstract

**Background:**

The emergence of drug-resistant tuberculosis (DR-TB) has been a major obstacle to global tuberculosis control programs, especially in developing countries, including Ethiopia. This study investigated drug resistance patterns and associated mutations of *Mycobacterium tuberculosis* Complex (MTBC) isolates from the Amhara, Gambella, and Benishangul-Gumuz regions of Ethiopia.

**Methods:**

A cross-sectional study was conducted using 128 MTBC isolates obtained from patients with presumptive tuberculosis (TB). Phenotypic (BACTEC MGIT 960) and genotypic (MTBDR*plus* and MTBDR*sl* assays) methods were used for drug susceptibility testing. Data were entered into Epi-info and analyzed using SPSS version 25. Frequencies and proportions were determined to describe drug resistance levels and associated mutations.

**Results:**

Of the 127 isolates recovered, 100 (78.7%) were susceptible to four first-line anti-TB drugs. Any drug resistance, polydrug resistance, and multi-drug resistance (MDR) were detected in 21.3% (27), 15.7% (20), and 15% (19) of the isolates, respectively, by phenotypic and/or genotypic methods. Mono-resistance was observed for Isoniazid (INH) (2, 1.6%) and Streptomycin (STR) (2, 1.6%). There were two genotypically discordant RIF-resistant cases and one INH-resistant case. One case of pre-extensively drug-resistant TB (pre-XDR-TB) and one case of extensively drug-resistant TB (XDR-TB) were identified. The most frequent gene mutations associated with INH and rifampicin (RIF) resistance were observed in the *katG MUT1* (S315T1) (20, 76.9%) and *rpoB* (S531L) (10, 52.6%) genes, respectively. Two MDR-TB isolates were resistant to second-line drugs; one had a mutation in the *gyrA MUT1* gene, and the other had missing *gyrA WT1*, *gyrA WT3*, and *rrs WT1* genes without any mutation.

**Conclusions:**

The detection of a significant proportion of DR-TB cases in this study suggests that DR-TB is a major public health problem in Ethiopia. Thus, we recommend the early detection and treatment of DR-TB and universal full first-line drug-susceptibility testing in routine system.

## Introduction

Tuberculosis (TB) is a curable and preventable disease with a broad host range, caused by the *Mycobacterium tuberculosis* Complex (MTBC) [1, 2]. It is the 13^th^ leading cause of death and the second leading cause of mortality from single infectious agents globally, next to the coronavirus disease (COVID-19) [3]. Despite a slow decline in incidence and the availability of effective treatment regimens, TB remains a major public health problem globally with 10.6 million cases in 2021 [4, 5]. According to a World Health Organization (WHO) report, Ethiopia is among the 30 high TB burden countries in the world, with an estimated incidence of 119 TB cases per 100,000 population in 2021 [5].

Drug-resistant TB (DR-TB) remains a major public health problem and a barrier to global TB control programs, especially in developing countries, such as Ethiopia. In 2021, WHO estimated 450,000 new cases of rifampicin-resistant TB (RR-TB)/MDR-TB globally, with MDR-TB prevalence rates of 3.6% in new cases and 18% in previously treated cases. Ethiopia has an estimated incidence of 1.5% MDR/RR-TB cases per 100,000 population, an estimated 1,800 MDR-TB cases annually, and reported 4 laboratory-confirmed XDR-TB cases in 2021 [5].

Early detection of DR-TB is recommended by the WHO as a global TB control strategy to prevent the spread of the disease [6, 7]. In recognition of this, the WHO has recommended universal access to drug-susceptibility testing (DST) [7]. However, most DR-TB cases remain undetected and consequently, continue to spread in the community [4, 8]. In addition, the burden of DR-TB in resource-limited settings is often assessed through periodic surveys [9]. This may be because most developing countries are unable to implement a continuous drug-resistance surveillance system due to technical and resource constraints [4, 8]. Consequently, the global incidence of DR-TB is viewed as an estimate, which implies that the actual burden might be underestimated [4, 9].

Unlike previous studies of TB drug resistance in the Amhara region [10–15], our study was conducted in multiple healthcare facilities located in different zones of the region. In addition, there are limited data on TB drug resistance in the Gambella and Benishangul-Gumuz regions [16, 17] and it would be useful to measure the levels of TB drug resistance in these specific areas. Therefore, the current study investigated drug resistance patterns and drug resistance-associated mutations of the MTBC isolates obtained from the Amhara, Gambella, and Benishangul-Gumuz regions of Ethiopia.

## Methods

### Study area

The study was conducted on MTBC isolates from the OMNIgene^®^ SPUTUM evaluation study in three regions of Ethiopia; Amhara, Gambella, and Benishangul-Gumuz (unpublished data). Ten hospitals with a high volume of patients were selected for the study; Assosa General Hospital, Ataye Hospital, Borumeda Hospital, Debre Birhan Referral Hospital, Debre Tabor General Hospital, Debre Markos Referral Hospital, Finote Selam Hospital, Gambella Hospital, Gonder University Hospital, and Metema Hospital.

### Study design and period

**Fig 1:**
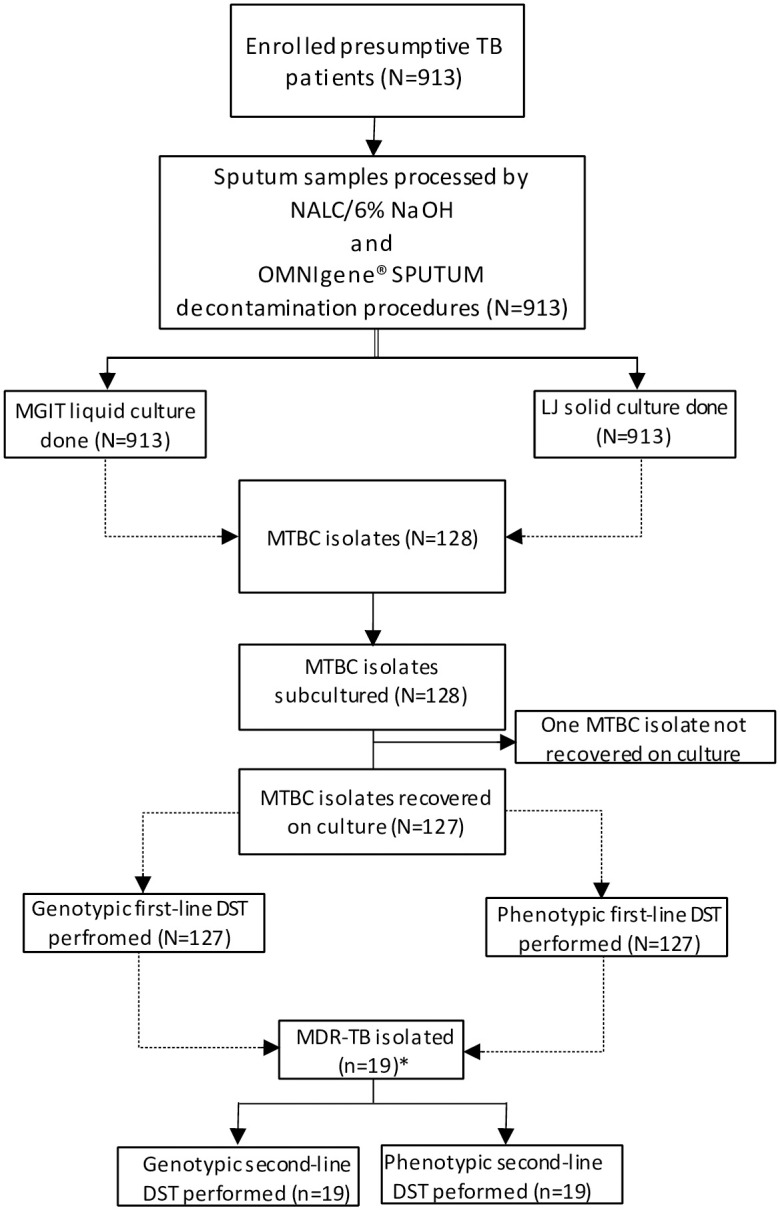
Flow chart showing the laboratory procedures of the study.

This cross-sectional study was conducted between January 2019 and March 2020 on 128 MTBC isolates. These isolates were obtained from participants enrolled between September 2017 and December 2018 for the OMNIgene^®^ SPUTUM evaluation study (Fig 1). The OMNIgene^®^ SPUTUM project included 913 presumptive TB patients aged ≥5 years with signs and symptoms of pulmonary TB, including cough for >2 weeks, fever, night sweats, unexplained weight loss or failure to gain weight, and history of contact with a TB patient per national guideline. All the enrolled participants provided written informed consent/assent. Participants were excluded if they were unable to provide written informed consent/assent, had only signs & symptoms of extra-pulmonary TB, and had received TB treatment within 6 months. Sputum samples were collected from patients who consecutively visited the selected healthcare facilities. Primary culture isolation was performed at the Amhara Public Health Institute (APHI) and Ethiopian Public Health Institute (EPHI). A total of 127 MTBC isolates recovered after subculturing were included in this current study. One isolate did not recover and was therefore excluded from the study.

### Laboratory methods

#### Mycobacterial subculturing

Mycobacterial identification was conducted on positive cultures, including microscopy (acid-fast bacilli check), blood agar inoculation (contamination check) and SD Bioline TB Ag MPT64 (Standard Diagnostics Inc., Kyonggi, South Korea) (MTBC identification). MTBC isolates were kept at -80°C at EPHI. Frozen MTBC isolates were thawed at room temperature and subcultured in MGIT liquid medium to perform phenotypic and genotypic DST [18]. Briefly, frozen MTBC isolates were thawed, vigorously vortexed, and then diluted with sterile saline (1:100 dilution). Aseptically, 0.8 ml growth supplement was added to 7 ml modified Middlebrook 7H9 MGIT broth. Finally, 0.5 ml of MTBC suspension (1:100) was inoculated into MGIT broth and incubated in the MGIT 960 instrument at 37°C. Growth was monitored for a maximum of 42 days [18].

#### Phenotypic drug-susceptibility testing

Phenotypic drug susceptibility tests (pDSTs) for first- and second-line anti-TB drugs were performed using the BD BACTEC MGIT 960 system following the manufacturer’s instructions and WHO’s recommendations [18, 19]. The final concentrations of the first-line anti-TB drugs (BD) were as follows: streptomycin (STR) [1.0 μg/ml], isoniazid (INH) [0.1 μg/ml], rifampicin (RIF) [1.0 μg/ml], ethambutol (EMB) [5.0 μg/ml] and pyrazinamide (PZA) [100 μg/ml]. While the second-line anti-TB drugs (Sigma) were Amikacin (Am) [1.0 μg/ml], Capreomycin (Cm) [2.5 μg/ml], Clofazimine (Cfx) [1.0 μg/ml], Ethionamide (Eto) [5.0 μg/ml], Kanamycin (Km) [2.5 μg/ml], Lenizolide (Lz) [1.0 μg/ml], Levofloxacine (Lvx) [1.0 μg/ml], Moxifloxacin (Mfx) [0.25 μg/ml] and Ofloxacin (Ofx) [2.0 μg/ml].

#### Deoxyribonucleic acid (DNA) isolation

DNA of the MTBC isolates was extracted from positive MGIT broth using the Hain Lifescience GenoLyse kit for GenoType MTBDR*plus* and MTBDR*sl* assays [20, 21].

#### GenoType^®^ MTBDRplus and MTBDRsl assays

Genotypic identification of first- and second-line anti-TB drug resistance was performed using the Genotype^®^ MTBDR*plus* (version 2.0) and Genotype^®^ MTBDR*sl* (version 2.0) assays, respectively, following the manufacturer’s instructions [20, 21]. The procedure included DNA extraction, amplification of the target sequences using biotinylated primers (AM-A and AM-B), and DNA reverse hybridization. The results were interpreted according to WHO recommendations [22].

### Data and laboratory quality control

Participants’ demographic (age, sex) and clinical data (clinical history, previous exposure to first-line anti-TB drugs (>1 month), previous TB treatment outcome, contact history with a known TB patient, contact history with a known MDR-TB patient, coinfections such as diabetes mellitus (DM), and HIV) were collected by trained professionals using standard OMNIgene^®^ SPUTUM evaluation study forms. The study participants were evaluated clinically for diabetes, and those suspected to have DM were tested using fasting blood sugar. Additionally, HIV testing was conducted on blood samples following national guidelines. A sequential rapid testing algorithm was used: Wantai HIV 1/2 (Beijing Wantai Biological Pharmacy Enterprise Co., Ltd, Beijing, China) as a screening test, Uni-Gold HIV 1/2^™^ (Trinity Biotech, plc., Wicklow, Ireland) as a confirmatory test, and Vikia HIV 1/2 (bioMérieux, SA, F-69280 Marcy l’Etoile, France) as a tie-breaker test. Participants with a nonreactive result on the screening test were considered HIV negative, while those with reactive screening tests underwent confirmatory testing. Those with reactive results on both tests were classified as HIV positive. Clinicians collected chest X-ray (CXR) findings as “indicative X-ray findings for TB”. These include parenchymal infiltrates, hilar adenopathy, pleural effusion, upper zone consolidation with ipsilateral hilar enlargement (lymphadenopathy), cavity lesions, nodular or fibrotic (reticular) densities, and opacification of airspaces within the lung parenchyma. Data were checked for completeness and consistency, and any missing or inconsistent data were resolved with focal person of the sites. Data were entered in duplicate on a pre-formatted Epi-info data sheet. All MTBC isolates identified by the APHI culture laboratory were transported to the EPHI in a cold chain and stored in a freezer (-80°C). All laboratory tests were performed by trained and competent professionals according to standard protocols (manufacturer’s instructions) and appropriate negative and positive controls were used. Molecular grade water and *Mycobacterium tuberculosis* H37Rv (ATCC 27294) were used as the negative and positive controls, respectively, in every batch of genotypic DST. Only *Mycobacterium tuberculosis* H37Rv (ATCC 27294) was included as a positive control in every batch of phenotypic DST.

### Data analysis

Demographic and clinical data were collected using a well-structured clinical data collection form, entered into Epi-info 2011 version 3.5.4, and analyzed using SPSS version 25. Descriptive analysis; frequencies and proportions were used to describe participant demographics, clinical profiles, patterns of resistance to first- and second-line anti-tuberculosis drugs, and mutations associated with drug resistance. The results are presented in tables and figure.

### Ethical considerations

The study received ethical clearance from Ethiopian Public Health Institute Institutional Review Board (EPHI-IRB-011-2017). We used stored mycobacterial isolates that were obtained from participants who had provided written informed consent/assent prior to participation. All adult participants provided written informed consent. For children aged <12 years, parents or legal guardians provided written informed consent. For children between 12 and 18 years of age, written informed assent was obtained. All participants diagnosed with TB were treated following the national guidelines. All participants’ information and results were kept confidential, and the dataset did not contain any personal identifying information about the participants; instead, unique study identification codes were used for the study.

## Results

### Demographic and clinical characteristics of study participants

A total of 913 sputum samples were collected from each presumptive TB patient, of which 128 (14.02%) were found to be MTBC positive by culture. Only 127 MTBC were recovered during subculturing and used for final data analysis. Of these, 4 (3.15%) were obtained from children and 123 (96.85%) were adults. The majority (61.4%) were male participants. The mean age was 31 years (SD ± 12.29). Ninety-nine (78.0%) isolates were obtained from the new cases. Majority of isolates were obtained from participants who presented with more than one TB signs and symptoms (94.5%) and had indicative chest X-ray findings suggestive of TB (70.9%). Twenty-three isolates (18.1%) were from participants living with HIV, while one was from participant with diabetes mellitus (Table 1).

**Table 1:** Demographics and clinical characteristics of culture-positive participants in the Amhara, Gambella, and Benishangul Gumuz regions of Ethiopia, April 2017 to December 2018 (N = 127).

Variables	Frequency (n)	Percentage (%)
**Region**		
Amhara	99	78.0
Gambella	14	11.0
Benishangul Gumuz	14	11.0
**Age-group**		
<15 years	4	3.1
15–24 years	39	30.7
25–34 years	46	36.2
35–44 years	17	13.4
45–54 years	10	7.9
≥55 years	11	8.7
**Sex**		
Male	78	61.4
Female	49	38.6
**Signs and symptoms of TB**		
Fever	115	90.6
Chronic cough for more than 2 weeks	126	99.2
Night sweating	106	83.5
Weight loss	121	95.3
Indicative chest X-ray finding for TB	91	71.7
**Contact history with known TB patient**		
Yes	29	22.8
No	98	77.2
**Previous history of TB/MDR TB**		
New case	99	78.0
Relapse case	17	13.4
Treatment after failure	5	3.9
Treatment after loss to follow-up	6	4.7
**Diabetes mellitus (DM)**		
Yes	1	0.8
No	126	99.2
**HIV status**		
Positive	23	18.1
Negative	104	81.9

### Overall drug resistance patterns of MTBC isolates

Of the 127 isolates, any drug resistance, MDR, and polydrug resistance were observed in 27 (21.3%), 19 (15%), and 20 (15.7%) isolates, respectively, using phenotypic and/or genotypic methods. Overall, 19 isolates (15%) were RIF resistant and 26 (20.5%) were INH resistant. There were two discordant RIF resistant cases and one INH resistant case. All discordant cases were genotypically but not phenotypically resistant (Tables 2 and 4). Additionally, one pre-XDR-TB case and one XDR-TB case were identified (Tables 3 and 4).

**Table 2:** Phenotypic drug resistance pattern of *M*. *tuberculosis* Complex isolates for first-line anti-TB drugs in the Amhara, Gambella, and Benishangul Gumuz regions of Ethiopia (N = 127).

The pattern of drug resistance	New cases (n = 99), n (%)	Retreatment cases (n = 28), n (%)	Total cases, n (%)
**Resistance**			
Any drug-resistance	9 (9.1)	18 (64.3)	27 (21.3)
Susceptible (4 tested drugs)	90 (90.9)	10 (35.7)	100 (78.7)
**Resistance to individual drugs**			
STR	7 (7.1)	13 (46.4)	20 (15.7)
INH	7 (7.1)	18 (64.3)	25 (19.7)
RIF	3 (3)	14 (50)	17 (13.4)
EMB	2 (2)	9 (32.1)	11 (8.7)
**Mono-resistance**			
Mono STR	2 (2)	-	2 (1.6)
Mono INH	2 (2)	-	2 (1.6)
Mono RIF	-	-	-
Mono EMB	-	-	-
**Multi-drug resistance**	3 (3)	14 (50)	17 (13.4)
**Poly-drug resistance**	5 (5.1)	15 (53.6)	20 (15.7)
**Resistance to all tested drugs**	2 (2)	6 (21.4)	8 (6.3)

EMB: ethambutol; INH: isoniazid; RIF: rifampicin; STR: streptomycin; “-”: not identified; Retreatment cases: relapse case, treatment after failure, and treatment after loss to follow-up.

**Table 3:** Phenotypic drug-resistance pattern of multidrug-resistant *M*. *tuberculosis* Complex isolates for second-line anti-TB drugs and PZA in the Amhara, Gambella and Benishangul Gumuz regions of Ethiopia (n = 19).

Second-line drug-resistance pattern	New cases (n = 3), n (%)	Retreatment cases (n = 16), n (%)	Total cases, n (%)
**Resistance**			
Any second-line drug-resistance	-	6 (37.5)	6 (31.6)
Susceptible to all second-line drugs tested	3 (100)	10 (62.5)	13 (68.4)
Resistance to PZA	2 (66.7)	14 (87.5)	16 (84.2)
**Resistance to individual second-line drugs**			
Am	-	-	-
Cm	-	-	-
Eto	-	5 (31.3)	5 (26.3)
Km	-	1 (6.3)	1 (5.3)
Ofx	-	1 (6.3)	1 (5.3)
Cfx	-	1 (6.3)	1 (5.3)
Lvx	-	1 (6.3)	1 (5.3)
Lz	-	1 (6.3)	1 (5.3)
Mfx	-	1 (6.3)	1 (5.3)
**Mono drug-resistance**			
Mono Eto	-	4 (25)	4 (21.1)
Mono Km	-	1 (6.3)	1 (5.3)
**Two or more second-line anti-TB drugs resistance**			
Eto + Ofx + Cfx + Lvx + Lz + Mfx (XDR-TB)	-	1 (6.3)	1 (5.3)

Am: Amikacin; Cm: Capreomycin; Cfx: Clofazamin; Eto: Ethionamide; Km: Kanamycin; Lvx: Levofloxacin; Lz: Linezolid; Mfx: Moxifloxacin; Ofx: Ofloxacin; PZA: Pyrazinamide; “-”: Not identified.

**Table 4:** Mutations associated with isoniazid, rifampicin, fluoroquinolones, and second-line injectable drug-resistant *M*. *tuberculosis* Complex isolates.

Anti-TB drugs	No. of resistant isolates	The pattern of gene mutations wild type/mutant	Amino acid change	Frequency (%)
Isoniazid	26	*ΔkatG WT/ katG MUT1*	S315T1	20 (76.9)
		*ΔkatG WT/katG MUT2*	S315T2	2 (7.7)
		*ΔkatG WT*/ND	-	1 (3.8)
		*ΔinhA WT/inhA MUT1*	C15T	3 (11.5)
Rifampicin	19	Δ*rpoB WT8/rpoB MUT3*	S531L	10 (52.6)
		Δ*rpoB WT7/rpoB MUT2A*	H526Y	3 (15.8)
		Δ*rpoB WT3 and rpoBWT4*/*rpoB MUT1*	D516V	2 (10.5)
		Δ*rpoB WT7*/ND	-	2 (10.5)
		Δ*rpoB WT3 and rpoBWT4*/ ND	-	1 (5.3)
		No missing *rpoB WT*/*rpoBMUT3*	-	1 (5.3)
Fluoroquinolones and second-line injectable drugs	2	*ΔgyrA WT2/gyrA MUT1*	-	1 (50)
		*ΔgyrA WT1 and gyrA WT3/* ND and *Δrrs WT1/* ND	G88A/G88C	1 (50)

Δ, deletion; MUT, mutant; ND, no mutation detected at the mutant probe; “-”, not identified.

### Phenotypic drug resistance patterns of MTBC isolates for first-line anti-TB drugs

First-line phenotypic DST was performed on 127 isolates. One hundred (78.7%) isolates were found to be fully susceptible to all the first-line anti-TB drugs tested. Any drug resistance was detected in 21.3% of isolates. The resistance rates to STR, INH, RIF, and EMB were 15.7%, 19.7%, 13.4%, and 8.7%, respectively. Mono-resistance was detected only in INH (1.6%) and STR (1.6%) cases. The prevalence of MDR among new and retreatment cases was 3% and 50%, respectively. Phenotypically, the MDR rate was 13.4% (17). Eight (6.3%) isolates were resistant to all drugs tested (Table 2).

### Phenotypic drug resistance patterns of MTBC isolates for second-line anti-TB drugs

Phenotypic DST to second-line anti-TB drugs and pyrazinamide (PZA) was performed for MDR-TB isolates (n = 19). Thirteen (68.4%) isolates were found to be fully susceptible to the nine tested second-line anti-TB drugs. Eto resistance was observed in 5 (26.3%) isolates, of which 4 were Eto mono-resistant. Km resistance was detected in one isolate (5.3%), which was also Km mono-resistant. Pre-XDR-TB was detected in one MDR isolate. One MDR isolate was found to be XDR-TB, which was resistant to Cfx, Lvx, Lz, Mfx, and Ofx. Interestingly, 16 MDR isolates (84.2%) were resistant to PZA (Table 3).

### Genotypic drug resistance patterns of MTBC isolates

Of the 127 isolates, 79.5% were genotypically characterized as susceptible to both RIF and INH. Isoniazid resistance was detected in 26 isolates (20.5%), of which 7 were INH resistant only. RIF resistance was detected in 19 (15%) isolates. All RIF-resistant isolates were also resistant to INH with genotypic DST and were MDR-TB (Table 4).

Among 19 MDR isolates, 89.5% were susceptible to fluoroquinolones and second-line injectable anti-TB drugs. Two MDR-TB isolates (10.5%) from previously treated TB patients were resistant to fluoroquinolones. One of these isolates was identified as XDR-TB by phenotypic DST using the latest WHO definitions. In addition, resistance to second-line injectable drugs was detected in one of these isolates by genotypic DST (Table 4).

### Mutations associated with drug resistance

Among 26 INH-resistant isolates, the predominant mutations were detected in *katG MUT1* gene with missing wild-type *katG* (amino acid change of S315T1) (76.9%), one of which was discordant INH-resistant case. Two (7.7%) had mutations in *katG MUT2* gene with missing wild-type *katG* (amino acid change of S315T2) and one (3.8%) had missing *katG WT* gene. Also, 3 (11.5%) had mutations in the *inhA MUT1* gene (promoter region) with missing wild-type in the *inhA* (amino acid change of C15T), of which two were INH mono-resistant cases (Table 4).

Among 19 RIF-resistant isolates, the most common mutations were the *rpoB MUT3* gene with missing wild-type in the *rpoB WT8* (amino acid change of S531L) (52.6%). Three isolates (15.8%) had mutations in the *rpoB MUT2A* gene with missing wild-type in the *rpoB WT7* (amino acid change of H526Y). Two (10.5%) had mutations in the *rpoB MUT1* gene with missing wild-type in both the *rpoB WT3* and *rpoB WT4* genes (amino acid change of D516V). Two (10.5%) had no mutations in the *rpoB* gene with missing wild-type in the *rpoB WT7*, of which one was a discordant RIF-resistant case. One (5.3%) had missing wild-type in both the *rpoB WT3* and *rpoB WT4* genes with no mutations in the *rpoB* gene and was also a discordant RIF-resistant case. One (5.3%) had mutations in the *rpoB MUT3* gene while not missing wild-type in the *rpoB* gene (Table 4).

Two MDR-TB isolates showed resistance in the genotypic assay. One had a mutation in the *gyrA MUT1* gene and a missing wild-type in the *gyrA WT2* with no known amino acid change and was identified as XDR-TB by phenotypic DST. In addition, one MDR isolate had no mutation while it had a missing wild type in the *gyrA WT1*, *gyrA WT3*, and *rrs WT1* genes, with an amino acid change of G88A/G88C and was phenotypically resistant to Km (Table 4).

## Discussion

In this study, we investigated the drug resistance patterns of MTBC isolates from the Amhara, Gambella, and Benishangul-Gumuz regions of Ethiopia and looked for drug resistance conferring mutations. This study identified a high proportion of DR-TB, including MDR, pre-XDR, and XDR-TB. The study also found mono-resistance cases to isoniazid and streptomycin. Moreover, INH and RIF drug resistance showed the most frequent mutations in *katG MUT1* (S315T1) and *rpoB* (S531L), respectively. FLQ resistance was associated with mutations in the *gyrA* gene.

The study showed that 21.3% of *M*. *tuberculosis* isolates were resistant to any of the first-line anti-TB drugs tested, using phenotypic DST. Similarly, other studies reported relatively comparable results of 20.2% and 23.3% [10, 23]. However, a higher resistance rate was reported from Southwestern Ethiopia (58.6%) [24]. In contrast, a lower resistance proportion was reported from Northwest Ethiopia (16.1%), Southern Ethiopia (11.1%), and Addis Ababa (13.3%) [17, 25, 26]. These variations might be due to differences in the clinical and sociodemographic characteristics of patients.

The highest rate of resistance was found for INH (19.7%). Higher resistance to INH compared to other first-line drugs was also reported in other studies in the country [17, 24]. In particular, up to 51.4% INH resistance was reported from Southwest Ethiopia [24]. This high INH resistance might be due to naturally high mutation [27] and the use of INH as a prophylactic drug for several years by the country’s TB control program for contacts of TB, latent TB, and HIV/AIDS patients [28, 29]. Thus, the detection of high INH resistance in this and previous Ethiopian studies could imply the need for full first-line phenotypic DST for all TB patients in the country so that they can be detected early and treated promptly with appropriate drugs.

In this study, mono-resistance was low and found only for INH and STR, with a rate of 1.6% each. However, higher INH mono-resistance (8.7%) was reported from elsewhere [11]. In addition, a previous study conducted in Ethiopia reported a higher INH or STR mono-resistance [23], which is inconsistent with our finding. Although mono-resistance was low in our study, more study is needed to better understand its prevalence because of the prolonged use of INH and STR drugs in the country’s TB control program. Besides, full drug susceptibility testing for all bacteriologically confirmed TB patients should be performed routinely to avert the development of additional drug resistance from INH mono-resistant cases, including its progression to MDR-TB.

The study also showed MDR-TB prevalence of 15%. However, a much higher prevalence rate (28%) was reported from Southwestern Ethiopia [30] and lower rates were reported by other studies in Benishangul-Gumuz (2.4%) [17], and in central Ethiopia (1.2%) [23]. However, caution is needed in interpreting these differences, as the data from Benishangul-Gumuz [17] included isolates obtained only from resource-limited region and its surroundings, which could explain the significant differences observed. Polydrug resistance was also detected in 15.7% of the isolates in this study as has been reported by previous studies [23, 25]. Polydrug resistance could negatively affect the treatment outcomes. In addition, polydrug resistance, which excludes RIF resistance, may remain undiagnosed as the country uses the Xpert MTB/RIF assay as the initial diagnostic test to identify TB and detect RIF resistance. This highlights the importance of ensuring universal access to full DST for all TB patients with bacteriological confirmation [7].

This study also identified pre-XDR-TB in one isolate and XDR-TB in one isolate, both from previously treated cases. In line with our findings, a previous study reported the detection of pre-XDR and XDR-TB cases, with previously treated cases more likely to develop DR-TB than new cases [15]. The pre-XDR cases in this study were due to resistance to fluoroquinolones, which is consistent with reports from other studies [15, 31]. The increasing use of fluoroquinolones to treat other bacterial infections, including respiratory, gastrointestinal, and urinary tract infections [32], may contribute to the emergence of resistance to these drugs. The pre-XDR and XDR-TB cases identified in this and previous studies in Ethiopia are alarming for the control program.

The most common INH resistance mutation identified was the *katG MUT1* gene (76.9%) with a missing wild-type, which is supported by a previous study [30]. The second most common INH resistance mutation was found in the *inhA MUT1* (promoter region) (11.5%) with missing wild-type in *inhA* gene. Interestingly, the two INH mono-resistant cases (new patients) showed this mutation; this mutation has also been reported previously [30, 31] and high-dose INH is likely to be effective in treating such cases. There were also INH-resistant isolates with unknown mutations (3.8%), where only the wild-type *katG* gene was missing. Similarly, a previous study has also reported INH resistance without an identified mutation [31]. The unknown mutations might be due to mutations outside *inhA* and *katG* gene regions, which warrants the need for further investigation.

The most common RIF resistance mutation was identified in the *rpoB MUT3* gene (52.6%), with missing wild-type in the *rpoB WT8* gene. Another study has also reported similar finding [26]. Furthermore, the second common RIF resistance mutation was identified in the *rpoB MUT2A* (15.8%), with missing *rpoB WT7* gene. Mutations in this gene have also been reported previously [31]. There were also RIF-resistant isolates that had no mutations, whereas either/and the *rpoB WT3*, *rpoB WT4*, and *rpoB WT7* were missing. In fact, such cases are not novel in Ethiopia, as previous studies had reported similar findings [13, 33]. These cases can be a result of mutations outside the 81bp regions of the *rpoB* gene.

Two MDR-TB isolates in this study were resistant to second-line anti-TB drugs. This finding is consistent with another study [26]. The study detected mutations associated with fluoroquinolones in two MDR-TB isolates and these fluoroquinolone resistances were associated with *gyrA* gene, which have been reported previously in other studies [15, 26]. Taken together, these findings highlight the importance of laboratories that can perform genotypic assays to diagnose TB and detect drug resistance, especially in resource-limited areas of the country, so that MDR-TB patients have timely access to drug resistance results for first- and second-line anti-TB drugs. In addition, these genotypic assays could help to understand the molecular epidemiology of MDR-TB in the absence of sequencing platforms.

Finally, the study results should be interpreted with the limitations in mind. The study was conducted on archived MTBC isolates as a continuation of another study. The number of culture-positive cases was low, and the isolates were not evenly distributed within the three study regions of Ethiopia. The GenoType^®^ MTBDRplus and MTBDRsl assays were used, but it should be noted that these methods cannot capture the full spectrum of drug resistance-conferring mutations. Nevertheless, our results provide important evidence on the drug resistance patterns and mutations associated with drug resistance in MTBC. Furthermore, in this study, we tested drug susceptibility to newly endorsed and repurposed second-line drugs such as Linezolid and Clofazimine, which is the first time in Ethiopia.

## Conclusions

The detection of a significant proportion of drug-resistant TB in this study suggests that DR-TB is a major public health problem in Ethiopia. Consequently, the country should focus on strategies that promote early detection and treatment of DR-TB and ensure universal access to full first-line DST for all TB patients.
